# Nuclear Export of Messenger RNA

**DOI:** 10.3390/genes6020163

**Published:** 2015-03-31

**Authors:** Jun Katahira

**Affiliations:** 1 Biomolecular Networks Laboratories, Graduate School of Frontier Biosciences, Osaka University, 1-3 Yamadaoka, Suita, Osaka 565-0871, Japan; E-Mail: katahira@anat3.med.osaka-u.ac.jp; Tel.: +81-6-6879-4606; Fax: +81-6-6879-4609; 2 Department of Biochemistry, Graduate School of Medicine, Osaka University, 2-2 Yamadaoka, Suita, Osaka 565-0871, Japan

**Keywords:** nucleo-cytoplasmic transport, Nxf1-Nxt1, mRNA, TREX complex

## Abstract

Transport of messenger RNA (mRNA) from the nucleus to the cytoplasm is an essential step of eukaryotic gene expression. In the cell nucleus, a precursor mRNA undergoes a series of processing steps, including capping at the 5' ends, splicing and cleavage/polyadenylation at the 3' ends. During this process, the mRNA associates with a wide variety of proteins, forming a messenger ribonucleoprotein (mRNP) particle. Association with factors involved in nuclear export also occurs during transcription and processing, and thus nuclear export is fully integrated into mRNA maturation. The coupling between mRNA maturation and nuclear export is an important mechanism for providing only fully functional and competent mRNA to the cytoplasmic translational machinery, thereby ensuring accuracy and swiftness of gene expression. This review describes the molecular mechanism of nuclear mRNA export mediated by the principal transport factors, including Tap-p15 and the TREX complex.

## 1. Introduction

Eukaryotic cells consist of various organelles that execute different activities to sustain a range of cellular functions. The largest among them is the cell nucleus, which is surrounded by the nuclear envelope (NE) and stores genetic information in the form of chromatin. Transcription of genes, processing of various RNAs and replication and repair of DNA occur in the nucleus, whereas translation of proteins exclusively takes place on the ribosomes in the cytoplasm. Due to this physical separation, messenger RNAs (mRNAs) must be exported to the cytoplasm where they direct protein synthesis, whereas proteins participate in the nuclear activities are imported into the nucleus. In addition, some types of RNAs reenter to the nucleus after being exported to the cytoplasm [1]. Therefore, nucleo-cytoplasmic transport of RNAs and proteins is essential for eukaryotic gene expression.

Among the various RNA species, mRNA is the most divergent in sequence, length, and structure. In addition, as recently demonstrated by proteomic analysis [2,3,4], mRNAs are associated with a myriad of proteins and exist as messenger ribonucleoprotein (mRNP) particles throughout their life [5,6]. The compositional complexity and the size of mRNPs are in contrast to those of other comparably small and simple RNAs, such as transfer RNAs (tRNAs) and microRNAs (miRNAs). Moreover, in the nucleus, precursor mRNAs (pre-mRNAs) undergo extensive processing including capping at the 5' end, splicing and polyadenylation at the 3' end, before being transported to the cytoplasm. Partially due to this unusual intricacy as transport cargoes, the nuclear export mechanism of mRNA is unique and distinct from those of the other small non-coding RNAs.

## 2. Nuclear Export of mRNA: A Brief Overview

Nuclear pore complexes (NPCs), which perforate the NE, are the main gateways through which RNAs and proteins are delivered to their proper destinations. The NPC is composed of approximately 30 distinct proteins that are collectively known as nucleoporins [7,8,9,10]. A subset of nucleoporins that line the central transport channel contains phenylalanine-glycine (FG)-repeat sequences, which emanate to the inside of the channel and form a dense hydrophobic meshwork that functions as a barrier limiting the improper exchange of soluble macromolecules between the nucleus and the cytoplasm [7,8]. Thus, nucleo-cytoplasmic transport of RNAs and proteins requires specific transport receptors to break this barrier.

The importin/karyopherin-β family of proteins comprise the prototypical transport receptor family that mediates nucleo-cytoplasmic movement of most proteins and small non-coding RNAs, such as tRNA, uridine-rich small nuclear RNA (UsnRNA), and miRNA [11,12,13,14,15]. These family members interact with the FG-repeats and various transport signals that are harbored in their cognate cargoes and direct them to the correct compartment. The small nuclear GTPase Ran dictates the direction of the transport mediated by the importin/karyopherin-β family of transport receptors by regulating the association and dissociation of the cargo-transport receptor complexes [11,12,13,14,15,16].

**Figure 1:**
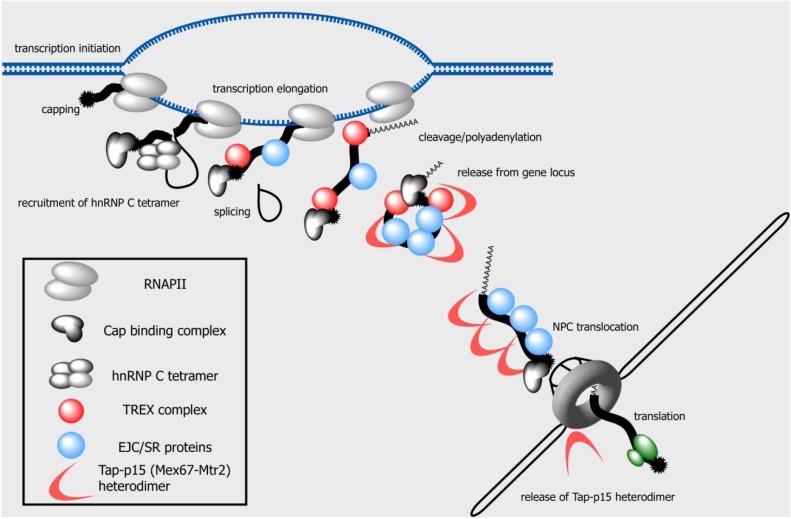
A brief overview of mRNA nuclear export. During transcription, protein factors required for capping, splicing and cleavage/polyadenylation are recruited to the nascent transcript, forming an mRNP. The 5' end of the mRNA is capped early in this process via an interaction between the capping enzyme and RNA polymerase II (RNAPII). Factors involved in splicing and cleavage/polyadenylation are also co-transcriptionally loaded onto the pre-mRNA (see also Figure 3). Measurement of the transcript length by the hnRNP C tetramer, which is important for allocating the transcript to the mRNA-specific processing and export pathway, could occur early during transcription. The TREX complex and a subset of the SR proteins, which are engaged in nuclear export, are recruited to the nascent mRNA via interactions with the transcription and processing factors. The nuclear export receptor Tap-p15 (Mex67-Mtr2 in yeast) in turn gains access to the mRNA via interactions with these factors as adaptors. The nuclear export receptor heterodimer facilitates the translocation of mRNPs through its interaction with FG-repeat containing nucleoporins. During the process of the nuclear mRNA biogenesis, the structure and the composition of the mRNP change drastically (see also Figure 4), and these alterations in the physicochemical properties also help the mRNP translocate through the NPC. The mRNA export factors are then dissociated from the mRNP by factors associated with the NPC to prevent the return of the mRNP to the nucleus. The exported mRNA then directs protein translation in the cytoplasm.

Nuclear export of mRNAs is a unique process that does not directly rely on the functions of the importin/karyopherin-β transport receptor family and Ran. Instead, it requires the evolutionarily conserved heterodimeric transport receptors Tap-p15 (also called Nxf1-Nxt1) in metazoans and Mex67-Mtr2 in yeast (Figure 1) [12,17]. A thermo sensitive mutant of *mex67* accumulates poly (A)^+^ RNA in the nucleus under the non-permissive temperature [18]. Human Tap and its orthologues from various metazoan species are also essential for cell viability, and nuclear accumulation of poly (A)^+^ RNA was observed upon down regulation of these genes in various organisms [19,20,21,22]. Although metazoan species harbor several Tap paralog genes, they are expressed only in specific tissues. Moreover, some of these proteins seem to have evolved to play other functional roles [23,24,25,26,27,28,29,30]. Thus, in general, structurally diverse mRNAs are exported by a single transport receptor.

**Figure 2:**
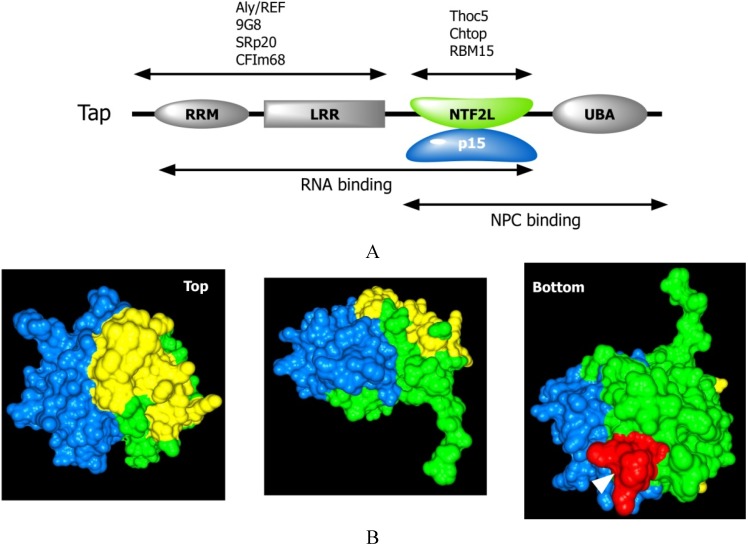
Structure and function of the principal mRNA export receptor Tap-p15. (**A**) Tap consists of an RNA recognition motif (RRM), leucine-rich repeat (LRR), nuclear transport factor 2-like (NTF2L) and ubiquitin-associated (UBA) domains. These domains are interconnected by flexible linkers (thin lines). Both the NTF2L and UBA domains contain FG-repeat-binding sites. Our recent analysis showed that the RNA binding activity of Tap is attributable to the RRM, LRR and NTF2L domains [50]. Adaptor proteins that bind to various domains of Tap are shown on top of the schema; (**B**) The structure of the NTF2L domain of Tap (green) complexed with p15 (blue). The surface of the NTF2L domain of Tap, which is critical for its RNA-binding activity, is shown in yellow. Note that the RNA- and the FG-repeat binding sites (an FG-repeat peptide in the complex is shown in red) are localized to opposing surfaces. The structural coordinate (accession number; 1JN5) was taken from the PDB database and displayed using the GRASP2 software [51].

Both Tap-p15 and Mex67-Mtr2 are RNA binding proteins, but they bind nonspecifically to RNA *in vitro* and are not able to distinguish different RNAs on their own [18,31,32]. To circumvent this problem, a series of mRNA-binding proteins participate in this process. The conserved transcription-export (TREX) complex, which consists of the THO subcomplex (composed of hHpr1, Thoc2, Thoc7, Thoc5, Thoc6 and hTex1 in mammals and Hpr1, Tho2, Mft1, Thp2 and Tex1 in yeast), Uap56 (Sub2 in yeast) and Aly/REF (Yra1 in yeast) plays an important role in selection of mRNAs by Tap-p15 and Mex67-Mtr2 [12,33,34,35,36,37]. The RNA-binding components of the TREX complex, including yeast Yra1 and mammalian Aly/REF, directly interact with the export receptor heterodimers, thereby functioning as adaptors (Figure 2A) [38,39]. In addition, in yeast, the serine-arginine rich (SR) proteins Npl3, Gbp2 and Hrb1, the latter two of which are associated with the TREX complex [40], and the mRNA binding protein Nab2 also interact with Mex67-Mtr2 and probably function as adaptors [41,42,43,44]. In mammalian cells, the SR proteins 9G8 and SRp20 [45], as well as numerous mRNA-binding proteins, have been proposed to play a similar role (Figure 2A) [22,46,47,48,49].

Recruitment of adaptor proteins to mRNPs is coupled with transcription and processing, causing mRNPs to be licensed to the mRNA-specific export pathway upon the completion of nuclear processing. Thus, transcription by RNA polymerase II (RNAPII) is a key determinant allocating mRNA to the appropriate export pathway. In addition, length is another important determinant that distinguishes mRNAs from UsnRNAs, both of which are transcribed by RNAPII in metazoans [52,53]. The heterogeneous nuclear ribonucleoprotein (hnRNP) C tetramer, which is an abundant nuclear mRNA binding protein, plays a critical role in this initial decision as a “molecular ruler” [53,54]. Recent studies concerning genome-wide profiles of RNAPII in yeast [55,56,57] suggest that size matters in nuclear export of mRNAs (see also Section 5.1).

It has been proposed that gene positioning to NPC-proximal site (known as “gene gating” [58]) is coupled to transcription initiation and nuclear export of mRNA in yeast. The yeast TREX-2 complex (Sac3-Thp1-Sem1-Sus1-Cdc31), which associates with the NPC through interaction with the SAGA (Spt-Ada-Gcn5-Acetyl transferase) transcriptional co-activator complex, plays important roles in this coupling and localizes a subset of transcriptionally active genes to NPCs [59,60,61,62,63,64]. Orthologues of the TREX-2 complex (GANP-PCID2-DSS1-ENY2-centrin) were also found in higher eukaryotes [63]. In mammals, in which transcription occurs deep inside the nucleus, the Sac3 orthologue GANP (germinal-center associated nuclear protein) binds to mature mRNPs via an interaction with Tap-p15 and chaperones them from the transcription sites to NPCs [65,66,67]. A recent report showed that the function of the mammalian TREX-2 is required for nuclear export of a subset of mRNA [68].

Several lines of evidence suggest that the mRNA export receptor directly interacts with an mRNA after its initial recognition via protein-protein interactions. This step has been shown to be regulated by post-translational modifications of the adaptor proteins [41,69]. Subsequently, the transport receptor facilitates translocation of the bound mRNA cargo through the NPC by directly interacting with the FG-repeat sequences of nucleoporins [32,70,71,72,73]. Upon translocation to the cytoplasm, the transport receptor is dissociated from the export complex to prevent the mRNA cargo from returning to the nucleus. This final step is facilitated by various factors, such as Gle1 and Dbp5, that are associated with the NPC [74,75,76,77].

## 3. Structure and Function of the mRNA-Specific Transport Receptor Heterodimer

Tap and Mex67 share a modular domain organization that comprises an RNA recognition motif (RRM) followed by leucine-rich repeat (LRR), NTF2-like (NTF2L) and ubiquitin-associated (UBA) domains (Figure 2) that are interconnected by unstructured flexible linkers [78]. The NTF2L domains of Mex67 and Tap tightly interact, respectively, with the small proteins Mtr2 and p15 [31,32]. Structural studies have revealed that this heterodimerization with the small partner proteins is crucial for maintaining the integrity of the NTF2L domain [79,80].

The NTF2L and UBA domains each contain a single FG-repeat-binding site. Although structurally unrelated, these domains bind to FG-repeat sequences in a manner similar to that of the importin/karyopherin-β family transport receptors [81]. These two FG-repeat-binding sites are essential for Tap-mediated mRNA export [82]. Interestingly, it has been shown that Tap derivatives containing two copies of either the NTF2L or UBA domain export mRNA less efficiently than the wild type protein [82], suggesting that these domains are not functionally equivalent and may have additional functional roles.

According to *in vivo* and *in vitro* assays, Tap-p15 and Mex67-Mtr2 directly interact with RNA. Although the intrinsic RNA-binding activity of the mRNA export receptors is weak, D-type retroviruses, such as Mason-Pfizer Monkey Virus (MPMV) and Simian Retrovirus type 1 (SRV-1), have evolved an effective way to specifically exploit Tap-p15 to export unspliced viral mRNA [83,84]. The constitutive transport element (CTE) is a structured, *cis*-acting RNA sequence that binds Tap-p15 with high affinity [85,86]. It has been thought that the amino-terminal half of Tap consisting of the RRM and LRR domains, which exhibits structurally and biochemically similar properties to the spliceosomal U2B'' and U1A' heterodimer, is sufficient for CTE binding. Indeed, a structural analysis has shown that the two domains extensively interact with the CTE [87,88]. However, our recent analysis revealed that the NTF2L domain also functions as an additional RNA binding platform that becomes apparent upon heterodimerization with p15 [50] (Figure 2B). Point mutations to the critical residues in the NTF2L domain, which are localized to the other side of the FG-repeat binding surface, severely reduced the CTE export activities. These data indicate that the binding through the NTF2L domain is functionally relevant for the CTE-driven mRNA export. Thus, the three domains of Tap; *i.e.*, the RRM, LRR and NTF2L domains, participate in RNA recognition (Figure 2A). The RNA binding activity of Mex67 of the budding yeast and the thermophilic fungus *Chaetomium thermophilum* is also attributable to the same domains, suggesting that the way by which the mRNA export receptors recognize cargo mRNAs is evolutionarily conserved [50,89]. Moreover, structural analysis of a Tap fragment containing the three RNA binding domains complexed with p15 revealed that the Tap-p15 forms an intimate domain-swapped dimer [90]. Intriguingly, RNA- and FG-repeat binding domains are arranged on the opposite faces in the dimer, thus efficiently interacting with the two-fold symmetrical structure of CTE-RNA and the FG-repeat containing nucleoporins. It is conceivable that the formation of the symmetrical RNA binding platform may promote the FG-repeat binding and accelerate the NPC translocation of CTE containing mRNA.

## 4. Bulk Cellular mRNA Recognition through mRNA-Binding Adaptor Proteins

To select bulk cellular mRNAs, Tap-p15 and Mex67-Mtr2 exploit a series of adaptor proteins. Yra1, the RNA-binding component of the TREX complex, is an essential adaptor protein in yeast and it directly binds to the amino-terminal domain of Mex67 through its arginine- and glycine-rich region [38,91]. In addition to Yra1, other adaptor proteins, such as Npl3 and Nab2, are also likely to mediate the recruitment of Mex67-Mtr2 to mRNAs [41,42,43]. Analysis of protein and RNA components of Nab2-bound mRNP revealed that Yra1 is co-purified with Nab2 and that the complex contains the bulk of yeast transcripts [43]. A recent transcriptome-wide PAR-CLIP (photoactivatable ribonucleoside-enhanced protein-RNA crosslinking and immunoprecipitation) analysis revealed that Mex67 binds mRNA without an apparent preference for specific RNA sequences. In contrast, the three adaptor proteins showed distinct crosslinking patterns, indicating that these factors bind to a unique spectrum of transcripts [92]. These observations, together with the previous data [93], suggest that multiple adaptor proteins enable the nuclear export of structurally divergent mRNA by a single transport receptor and that the three adaptor proteins could be grouped as general, *i.e.*, Yra1 and Nab2, and specific, *i.e.*, Npl3, with regard to their repertoire.

Aly/REF, an orthologue of Yra1, also interacts with the amino-terminal region of Tap [39]. In contrast to Yra1, which is essential for mRNA export in yeast, Aly/REF in metazoans is required but not essential for bulk cellular mRNA export [94,95]. These earlier observations have led to the hypothesis that an additional adaptor protein with a partially redundant function might participate in the recognition of bulk mRNAs in metazoans [81,94]. Thoc5, a metazoan-specific RNA-binding component of the TREX complex, interacts with the NTF2L domain of Tap, which completely overlaps with the RNA-binding platform described above [22]. We found that Tap mutants harboring mutations in either the RRM or the NTF2L domain exported mRNAs as efficiently as the wild type protein. Notably, mutations in both the RRM and NTF2L domains severely blocked the mRNA binding and export activities of Tap [50]. In addition, the nuclear export of bulk cellular mRNA was not severely affected in a human cell line exclusively expressing the NTF2L domain mutant of Tap. Importantly, the cell line exhibited a synthetic growth phenotype due to a severe mRNA export block when Aly/REF was knocked down [50]. Moreover, simultaneous knock down of Aly/REF and Thoc5 blocked the nuclear export of mRNA in the parental wild type cell line [50,96]. Taken together, these data suggest that, at least in mammalian cells, the TREX component Thoc5 could be the factor that cooperates with Aly/REF. Recent studies indicated that the nuclear export of only a subset of genes is impeded in mammalian cells under Thoc5 depleted condition [97,98,99]. This result suggests that the nuclear export of certain types of mRNAs is differentially dependent on the two principal adaptor proteins. Aly/REF has been shown to be involved in nuclear phosphoinositide signaling [100]. Recent data showed that phosphatidylinositol (3, 4, 5)-triphosphate (PIP3), a product of inositol polyphosphate multikinase (IPMK), is required for Aly/REF to selectively recognize RAD51 mRNA [101], indicating that the target mRNA recognition by the adaptor proteins is regulated. In addition, the range of adaptor proteins that have been identified in mammals to date may further expand the repertoire of Tap-p15, as has been proposed for the yeast adaptor proteins [92]. Moreover, a recent report indicated that a tissue specific adaptor protein complements the function of Aly/REF [102]. However, we still do not fully understand whether and how these different adaptor proteins participate in the nuclear export of various mRNAs. Thus, determination of the specificity of each adaptor and the relevance in the nuclear export of different mRNAs in mammals awaits further analysis.

## 5. Formation of Export Competent mRNPs

Soon after the initiation of transcription, an mRNA is coated with a multitude of proteins and thereby always exists as an mRNP. The protein components of an mRNP range from factors that participate in processing, packaging and nuclear export to those that function in the decay, translation and localization of the mRNP. Some of these components are released in the nucleus, while others accompany the mRNP to the cytoplasm. Thus, throughout life, the structure of an mRNP and the composition of its associated proteins are continually changing [5,6,103,104,105]. Moreover, many studies have suggested that the way how an mRNP is formed in the nucleus affects its fate in the cytoplasm [52,106,107,108,109,110,111].

### 5.1. Transcription-Coupled mRNP Formation

**Figure 3:**
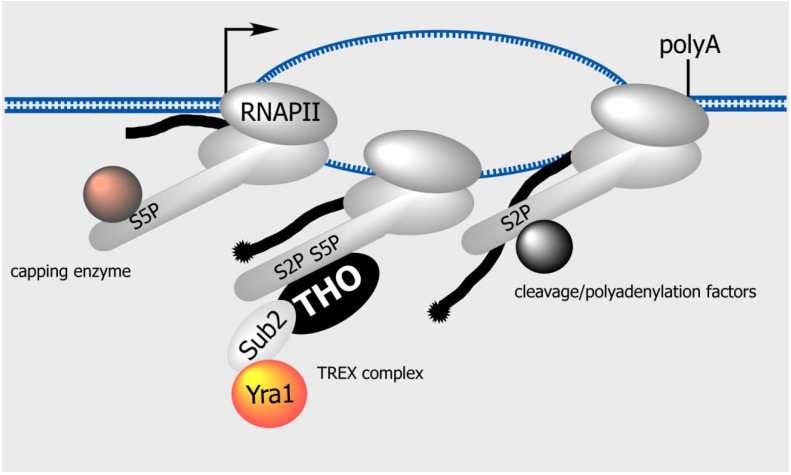
Co-transcriptional loading of the TREX complex. The carboxy-terminal domain (CTD) of RNAPII is subjected to transcription-cycle-specific modifications. Ser5 of CTD is phosphorylated when RNAPII is near the promoter. The capping enzyme specifically binds to the Ser5-phosphorylated CTD, allowing capping to occur early during transcription initiation. Ser2 phosphorylation gradually increases during transcription elongation and becomes dephosphorylated near the transcription termination site. Factors involved in cleavage/polyadenylation specifically bind to the Ser2-phosphorylated CTD. Recent data indicate that the THO subcomplex of the yeast TREX complex specifically and directly interacts with the Ser2/Ser5-diphosphorylated CTD [116].

The co-transcriptional processing of mRNAs, which is mediated by the carboxy-terminal domain (CTD) of the largest subunit of RNAPII [112,113,114,115], is important for appropriately directing transcripts to their mRNA-specific processing and export pathway (Figure 3).

The CTD consists of a highly conserved heptapeptide repeat: Y_1_S_2_P_3_T_4_S_5_P_6_S_7_, the numbers of which vary across species, ranging from 26 in yeast to 52 in humans. The repetitive sequence functions as a binding platform for a wide variety of nuclear factors involved in capping, transcription elongation, splicing and cleavage/polyadenylation. Transcriptional stage specific modification of the CTD determines the types of factors that associate with it. For example, S_5_ of the CTD is phosphorylated during transcription initiation and is rapidly dephosphorylated upon the transition to transcription elongation. Thus, S_5_ phosphorylation is high at the promoter region and persists at low levels throughout the gene body. The capping enzyme is specifically attracted to the S_5_ phosphorylated CTD, and, thus, the cap is added to the 5' end of mRNA co-transcriptionally during transcription initiation [112]. In contrast, S_2_ phosphorylation occurs later, increases during transcription elongation and decreases shortly after RNAPII reaches the transcription termination site. Factors that act later in the transcription cycle, such as splicing, cleavage and polyadenylation, selectively interact with an S_2_ or S_2_/S_5_ phosphorylated CTD. Earlier studies have revealed that the localization pattern of the yeast THO and TREX components on active loci, which increases from the 5' to the 3' region, resembles to that of S_2_-phosphorylated RNAPII [117,118]. However, the exact mechanism by which the THO/TREX complex is recruited co-transcriptionally has yet to be fully elucidated. A recent analysis in yeast revealed that the THO subcomplex specifically and directly interacts with the S_2_/S_5_-diphosphorylated CTD. Thus, the 5' to 3' increase in S_2_ phosphorylation directly mediates the recruitment of the TREX complex to the active genes [116]. The elongating chain of the nascent mRNA, to which the THO component, such as Tho2 binds, and the Prp19 complex, which also interacts with the elongating RNAPII and the THO/TREX complex, further stabilize the binding of the TREX complex to the active loci [119]. This transcription-coupled mechanism could also be in operation especially in intronless genes in metazoans. In fact, the TREX components, as well as the Prp19 complex and U2AF2, have been shown to associate with cytoplasmic accumulation region element (CAR-E), a 10-nt RNA sequence identified in a subset of naturally intronless mRNAs that promotes their nuclear export and stable accumulation in the cytoplasm [120].

It has recently been reported by different groups that the phosphorylation cycle of RNAPII CTD is very similar at all transcribed yeast genes [55,56,57]. They observed that following S_5_ phosphorylation, S_2_ phosphorylation occurs always approximately 600 bps away from the transcription start site, irrespective of the gene length. Therefore, it is conceivable that short and long genes can have different CTD phosphorylation levels at the transcription termination site; short genes tend to have higher levels of S_5_ and lower levels of S_2_ phosphorylated RNAPII [57]. The recruitment of factors that participate in pre-mRNA processing, including termination factors and mRNA-binding adaptor proteins, could be differently influenced by the CTD phosphorylation pattern. Consequently, the size of gene could affect nuclear export of the mRNA.

After being recruited to transcriptionally active genes, the mRNA-binding adaptor proteins Yra1 and Aly/REF in the TREX complex are transferred to the mRNA, where they then recruit the export receptors Mex67-Mtr2 and Tap-p15. This final step is proposed to be regulated by yeast Sub2 and metazoan Uap56, both of which serve as DExD/H-box-type RNA helicases in the TREX complex [121,122]. The ATP-bound, but not ADP-bound, Uap56 interacts with RNA and Aly/REF *in vitro*. The binding of RNA and Aly/REF then stimulates the intrinsic ATPase activity of Uap56, inducing the dissociation of Uap56 from the complex. Since Uap56 and Tap-p15 competitively bind to Aly/REF, the release of Uap56 from the complex allows Aly/REF to recruit Tap-p15 [123].

The TREX components are also recruited to the mRNA during the transcription termination via interactions with the cleavage/polyadenylation factors [124,125]. Synchronization of the export adaptor recruitment with pre-mRNA cleavage and polyadenylation is important to release the mRNP upon maturation and to prevent the unnecessary retention of mRNAs at gene loci, which may threaten genome stability [124,126].

### 5.2. Splicing-Coupled mRNP formation

**Figure 4:**
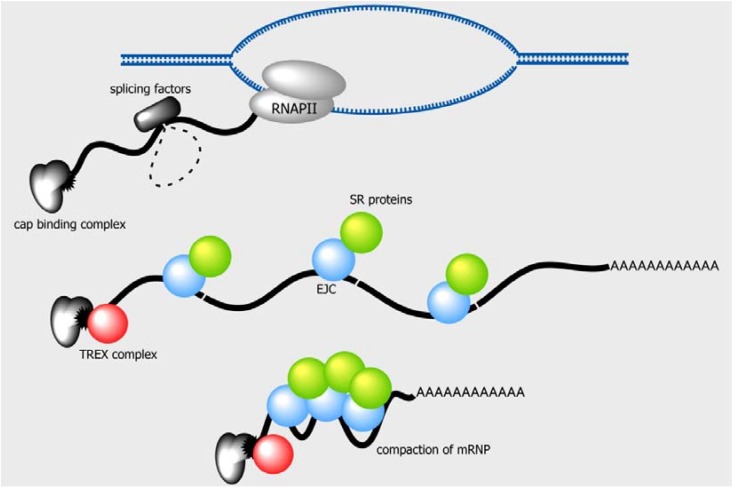
Splicing changes the structure and the composition of an mRNP. The exon-junction complex (EJC) and a subset of SR proteins are deposited on the mRNP upon splicing. Multimerization of the EJC along with the associated SR proteins promotes mRNP packaging and compaction. An earlier study has shown that splicing also recruits the TREX complex to the 5'-end of mRNA [130]. This compaction and the association of the nuclear export factors may promote the translocation of the mRNP through the NPC.

It has been known for years that splicing stimulates gene expression, but the step at which splicing acts has been elusive (see reviews [81,127,128] for a discussion of this topic). One compelling theory is that splicing promotes the nuclear export of mRNAs. Indeed, it has been shown that in metazoans, of which most genes harbor multiple introns, the TREX complex is recruited to mRNPs during splicing [129,130] (Figure 4). Uap56, a component of the metazoan TREX complex, was originally identified as an interaction partner of splicing factor U2AF2 [131]. In addition, splicing deposits SR proteins and the exon junction complex (EJC), a multiprotein complex that binds approximately 24 nucleotides upstream of each exon boundary onto the spliced mRNA [132,133] (Figure 4). In mammalian cells, the SR proteins (see the previous sections) and several EJC components interact with Tap-p15 [134,135,136] and, therefore, directly couple nuclear mRNA export to splicing. In contrast to metazoans, yeast Mex67-Mtr2 recognizes cargo mRNAs via the adaptors recruited co-transcriptionally to mRNP (see Section 5.1). Yeast mRNA binding adaptor proteins, such as Npl3, are co-transcriptionally recruited to mRNA and then, in turn, promote transcription elongation and splicing [137,138,139], linking splicing and nuclear export of mRNA. Thus, it is possible that the ways by which transcription and nuclear export are coupled are not the same among eukaryotic species, due, at least in part, to the abundance of introns in their genome.

### 5.3. Compaction of mRNPs during Processing

In addition to the recruitment of the nuclear mRNA export factors, processing drastically alters the structure and composition of mRNPs. Indeed, it has been reported that the lengths of purified mature mRNPs from yeast were considerably shorter than the expected lengths of the mRNAs within them, indicating that processed mRNPs are highly compacted [43]. The compaction of mRNPs is also supported by the results of an analysis of the diffusion coefficients of fluorescently labeled mRNAs in living mammalian cells [140] and of structural studies of Balbiani ring gene mRNPs in the salivary glands of *Chironomus tentans* [141]. In addition, *in vivo* imaging of labeled endogenous mRNA has revealed that the mRNA export process can be divided into three steps, *i.e.*, docking to, translocation through and release from the NPC, and that the translocation is not the rate-limiting step and proceeds very quickly (it takes only 5 to 20 ms), indicating that properly processed mRNP is prone to translocation [142,143].

Recent evidence has indicated a role of splicing in the structural alteration of mRNPs at the molecular level. A comprehensive analysis of the endogenous EJC protein and mRNA interactomes in human cells has revealed that the EJCs are present on the vast majority of exon-exon junctions and form high-molecular-weight multimers with their associated SR proteins [144] (Figure 4). The multimerization of EJCs that bind to various positions within a single mRNA could induce the compaction of the mRNP. Furthermore, the compaction of an mRNP may alter the proximity of various sites in a single mRNA, thus influencing mRNA processing patterns. An overall reduction in size due to compaction may also facilitate the ability of an mRNP to translocate through the NPC. Interestingly, both the EJC and SR proteins are rich in intrinsically disordered regions, which mediate the phase transition of mRNPs to hydrogels [145,146]. Changes in the physicochemical properties of mRNPs induced by the association of the EJC and SR proteins may also expedite the translocation of mRNPs through the hydrophobic milieu formed by the FG-repeat hydrogel within the central channel of the NPC [147,148,149,150,151].

### 5.4. Surveillance Mechanisms for mRNA Export

Any failure at each step during the biogenesis of mRNA results in production of faulty mRNP, which, in principle, threatens genome integrity and the normal proteome in the cell. To eliminate the functionally defective mRNP and ensure translational fidelity, cells have evolved multi-layered surveillance mechanisms. The translation-dependent quality control, which takes place in the cytoplasm, is the best-studied mechanism to degrade defective mRNAs and proteins deposited in mRNP in the nucleus play important roles (see [152] for a recent review). In addition, various nuclear surveillance mechanisms, which closely link to the mRNA export step, are in operation in the nucleus (see [36,153] for reviews). Immature mRNPs containing unspliced transcripts are retained in the nucleus by components of the spliceosome [154,155] and factors associated with the NPCs [156,157,158]. The yeast TREX-2 complex has also been shown to contribute to the retention of immature mRNP at the transcription site and nuclear periphery [159]. The retained mRNP is subjected to nuclear RNA decay activities for quality control. In yeast, the nuclear exosome, which mediates 3'–5' degradation of RNAs, with the aid of the TRAMP (Trf4/Air2/Mtr4 polyadenylation) complex comprises the major decay pathway, whereas decapping followed by 5'–3' degradation mediated by Dcp2 and the Rat1-Rai1 complex, functions as the minor pathway [36,153]. In addition, a yeast endoribonuclease Swt1, which transiently associates with the NPC, has been shown to participate in the degradation of defective mRNPs trapped at the nuclear periphery to avoid their cytoplasmic export and translation [160].

## 6. Conclusions and Perspectives

Extensive studies have greatly clarified the molecular mechanisms of mRNA export. Nuclear mRNA export is fully integrated into gene expression, and it proceeds with other elementary steps of gene expression. The TREX complex plays pivotal roles in the coupling of these processes through the extensive interaction networks with the factors involved in transcription, splicing, polyadenylation, and nuclear export.

mRNAs are associated with various proteins, including a variety of adaptor proteins. The inclusion of a diverse set of adaptor proteins within a single mRNP may increase its chance of being recognized by the transport receptor. Recruitment of multiple copies of transport receptors may also be advantageous for efficient transport of huge mRNPs, as has been suggested for ribosomal particles [161,162]. Alternatively, these adaptor proteins may enable the nuclear export of different mRNPs by a single transport receptor. As recently suggested, it is also possible that different adaptors function sequentially during the course of mRNP maturation [46]. However, due to technical difficulties, the protein compositions of individual mRNPs, as well as those of their intermediates still remain to be elucidated. Detailed analysis of the transcript-specific association of mRNA export factors, especially in mammalian cells, will certainly help answer these open questions.

While nuclear mRNA export is essential for eukaryotic cells, it is also crucial for certain pathogens, such as viruses that replicate in the host cell nucleus. As various studies have exemplified, the transport receptor Tap-p15 and the TREX components are exploited to transport viral mRNAs (for recent reviews see [163,164]). Although the details remain enigmatic, the mRNA export pathway may include various subroutes that are differentially dependent on particular adaptor proteins. Therefore, a more detailed dissection of the nuclear mRNA export pathway in mammalian cells will be beneficial not only to better understand the general gene expression mechanism, but also to provide information for more practical research applications, such as the development of anti-viral drugs.
